# Dynamic cytological and transcriptomic analyses provide novel insights into the mechanisms of sex determination in *Castanea henryi*

**DOI:** 10.3389/fpls.2023.1257541

**Published:** 2023-09-12

**Authors:** Guolong Wu, Xiaoming Tian, Qi Qiu, Yue Zhang, Xiaoming Fan, Deyi Yuan

**Affiliations:** ^1^ Key Laboratory of Cultivation and Protection for Non-Wood Forest Trees, Ministry of Education, Central South University of Forestry and Technology, Changsha, China; ^2^ Key Laboratory of Non-Wood Forest Products of State Forestry Administration, Central South University of Forestry and Technology, Changsha, China; ^3^ Hunan Botanical Garden, Changsha, China

**Keywords:** chestnut, cytokinin, floral organ identity genes, gene expression, regulatory network, weighted gene co-expression network analysis

## Abstract

*Castanea henryi* is a monoecious woody food tree species whose yield and industrialization potential are limited by its low female-to-male flower ratio. Here, the male flowers on the male inflorescence of *C. henryi* were converted to female flowers by triple applications of exogenous cytokinin (CK) (N-(2-chloro-4-pyridyl)-N’-phenylurea, CPPU). To study the role of exogenous CK in flower sex determination, cytological and transcriptomic analyses were performed on samples from the five stages after CK treatment. Cytological analysis showed that stage 3 (nine days after the last CK treatment) was the critical stage in the differential development of the pistil primordium and stamen primordium. On this basis, one key module and two modules with significant positive correlations with stage 3 were identified by weighted gene co-expression network analysis (WGCNA), combined with transcriptome data. The CK and GA biosynthesis- and signaling-related genes, three transcription factor (TF) families, and 11 floral organ identity genes were identified in the related modules. In particular, the TFs *WRKY47*, *ERF021*, and *MYB4*, and floral organ identity genes *AGL11*/*15*, *DEF*, and *SEP1* with large differences are considered to be critical regulators of sex determination in *C. henryi*. Based on these results, a genetic regulatory network for exogenous CK in the sex determination of flowers in *C. henryi* is proposed. This study contributes to the understanding of the role of CK in the sex regulation of flowers and provides new insights into the regulatory network of sex determination in *C. henryi.*

## 1 Introduction

Henry’s chestnut or Chinese chinquapin, *Castanea henryi* (Skam) Rehd. et Wils. (Fagaceae), is one of the most important tree species used in both timber and nut production in China. Due to its high ecological and economic value, it is widely planted and plays a major role in forestry production and economic development. The nuts of *C. henryi* are rich in nutrients, including starch, protein, fat, soluble sugar, and flavonoids (Ertürk et al., 2006), which are beneficial to human health and improve immunity, anti-arrhythmia, and lipid reduction (Morton et al., 2000; Yao et al., 2004). However, as a monoecious plant, the problem of having too many male flowers and too few female flowers has become one of the main problems restricting the development of the chestnut industry. Hence, it is urgent to study the molecular mechanism of sex determination in *C. henryi* flowers and explore techniques for artificially regulating the ratio of female flowers.

To increase the seed yield of monoecious plants, it is crucial to appropriately adjust the ratio of male to female flowers. Many phytohormones are involved in unisexual flower sex determination and developmental pathways, which play an indispensable role in organ differentiation and function at different stages of plant development (Ubeda-Tomas and Bennett, 2010). Generally, gibberellin (GA) promotes the formation of male flowers. In the monoecious plant *Buchloe dactyloides* (Nutt.) Engelm., GA treatment resulted in a higher proportion of male inflorescences in all three genotypes tested. However, when treated with paclobutrazol, a GA inhibitor, the three genotypes produced a higher proportion of female inflorescences, indicating the dual effect of GA-inducing males and inhibiting females (Yin and Quinn, 1995). In *Spinacia oleracea* L., the ratio of male to female flowers increased significantly when treated with 0–100 mg/L GA_3_ after the seedling stage, and this effect was enhanced with increasing GA_3_ concentration (El-Gizawy et al., 1992). In contrast, cytokinin (CK) is considered an active regulator of pistil development. In *Sapium sebiferum* (L.) Roxb., exogenous application of 6-benzylaminopurine or thidiazuron significantly promoted the development of female flowers and increased the number of fruits. Moreover, the feminization effect also affected the androecious genotype of *S. sebiferum*, which only produces male flowers (Ni et al., 2018). Similarly, CK had a significant female-promoting effect on sex differentiation in *Mercurialis annua* L. (Irish and Nelson, 1989) and *Vitis amurensis* Rupr. (Jun et al., 2002), and CK treatment could make flowers with a male genotype change to a female phenotype, subsequently flowering and fruiting. Nevertheless, most studies on the hormonal regulation of sex differentiation in higher plants have focused on the effects of hormones, and the molecular basis of the process is still poorly understood. Consequently, it is important to study the temporal and spatial expression processes of female and male flowers at the molecular level to reveal the hormonal regulatory mechanisms of sex determination in higher plants.

In our previous study, we demonstrated that CK and GA play pivotal roles as phytohormones in determining the sexual differentiation of flowers in *C. henryi* (Fan et al., 2017), Furthermore, when N-(2-chloro-4-pyridyl)-N’-phenylurea (CPPU), a type of CK, was sprayed at a concentration of 125 mg/L three times on *C. henryi* plants, a complete conversion of male inflorescence into a purely female inflorescence was observed. However, when sprayed once or twice, or at concentrations of 5 mg/L and 25 mg/L, only a partial transformation from male inflorescence to female inflorescence was observed (Wu et al., 2022). In the present study, all male flowers in the male inflorescence of *C. henryi* were transformed into female flowers by spraying exogenous CK three times. Accordingly, dynamic cytological and transcriptomic analyses were combined to investigate the mechanisms by which CK promotes the feminization of *C. henryi* flowers at the cellular and molecular levels. First, morphological and cytological analyses were used to observe the male inflorescences of *C. henryi* at different stages after treatment with CK (3, 6, 9, 12, and 15 days after the last CK treatment). Second, transcriptome sequencing was performed on male catkins of *C. henryi* treated with CK. Third, weighted gene co-expression networks were constructed by combining cytological and transcriptomic data to identify the gene co-expression modules. Finally, real-time quantitative polymerase chain reaction (RT-qPCR) was used to verify the expression of 15 key transcription factors (TFs) identified in this study. The present study improves our understanding of the molecular mechanism of floral sex determination in *C. henryi*, and provides an effective reference for the artificial regulation of female-to-male flower ratios in plants.

## 2 Materials and methods

### 2.1 Plant materials and treatments

The already existing plantation of the *C. henryi* cultivar ‘Huali 4’, with good growth and similar growth potential, was used in the present study. Trees were planted with 2.5 m × 3 m spacing and sourced from the Central South University of Forestry and Technology (28°10’ N, 113°23’ E) in Hunan Province, China. A stock solution of plant hormone was prepared by dissolving 125 mg of CPPU in 25 mL of absolute ethanol. Subsequently, the stock solutions were diluted with 1 L of distilled water to obtain working solutions with a concentration of 125 mg/L. Male inflorescences of six 12-year-old healthy *C. henryi* trees were treated with 125 mg/L CPPU during the first (March 28, 2022), second (April 4), and third weeks (April 11) after the buds sprouted for the first time (Wu et al., 2022), and water, in which 25 mL of ethanol had been dissolved, was used as a control.

### 2.2 Sample collection

After the third CK or water treatment, male inflorescences were collected at 3-day intervals (April 14, April 17, April 20, April 23, and April 26, 2022, described as stages 1–5, respectively). For each treatment, six branches were randomly selected to provide inflorescence samples. The 5^th^–10^th^ inflorescences, counted from the base of the branch, were collected as test samples.

For accuracy, each inflorescence was divided into lower, middle, and upper parts, named L (lower), M (middle), and U (upper), respectively. In practical terms, the lower end of the male inflorescence at stage 1 after CK treatment was described as T1L, and the lower end of the male inflorescence at stage 1 after water treatment was described as C1L. Some of the collected samples were rapidly frozen in liquid nitrogen and stored in a −80°C freezer for transcriptomic sequencing and RT-qPCR, and some were prefixed in Carnoy’s solution (acetic acid:ethanol = 1:3 v/v) for subsequent cytological analysis.

### 2.3 Optical microscope observation

The samples were pretreated with Carnoy’s solution for 4 h, followed by gas removal for 6 h, and then placed in 70% ethanol for 48 h at 4°C. Subsequently, the samples were dehydrated using an ethanol series, made transparent, and embedded in paraffin. Finally, the tissues were sliced to 9 *μ*m thickness using a Leica RM2235 rotary micrograph (Leica Camera AG, Solms, Germany), stained with hematoxylin-eosin, and observed and photographed under a light microscope (DMi8; Leica, Wetzlar, Germany) (Fan et al., 2015; Fan et al., 2017; Zhong et al., 2020; Qiu et al., 2023).

### 2.4 Transcriptome sequencing

The Trizol Reagent Kit (Invitrogen, Carlsbad, USA) was used to extract total RNA from the male inflorescences, and a UV spectrophotometer and NanoDrop 2000c spectrophotometer (Thermo Fisher, Waltham, MA, USA) were used to detect the concentration and purity of RNA. The sequencing libraries for each sample (a total of 90 cDNA libraries, represented by five collection times from three different parts in two different treatments and three biological replicates) were constructed by Wuhan Igenebook Biotechnology Co., Ltd. (Wuhan, China).

After raw data quality control was performed using FastQC (version: 0.11.5) (Andrews, 2010), the obtained high-quality clean reads were compared to the reference genome using HISAT2 (version: 2.0.1-beta) (Kim et al., 2015). After obtaining valid reads, the number of the aligned reads was counted using featureCounts (version: v1.6.0) (Liao et al., 2014) from the annotated files of the genome, and the standardized method fragments per kilobase of exon per million reads mapped (FPKM) (Mortazavi et al., 2008) was used to horizontally compare the expression difference between different samples of the same gene.

Genes with a fold change > 2 and false discovery rate < 0.05 were considered as differentially expressed genes (DEGs). All DEGs were searched against the NCBI non-redundant protein database (Nr) and annotated in detail based on Gene Ontology (GO) (Ashburner et al., 2000; Consortium, G.O, 2015) functions and Kyoto Encyclopedia of Genes and Genomes (KEGG) (Kanehisa and Goto, 2000; Kanehisa et al., 2021) pathways.

The weighted gene co-expression network analysis (WGCNA) R package (Langfelder and Horvath, 2008) was used to identify critical regulatory genes that responded to exogenous CK treatment. Similar modules were screened in the hierarchical tree using a dynamic tree cut procedure (merge cut height = 0.75, minimum module size = 30), with an adjacency matrix between different genes constructed, which was based on a threshold power of 8. The expression profile of module genes in each sample was represented by defining the module eigengene as the first principal component of the given module. Pearson correlation coefficient (PCC) with a Student’s t- test (*P* < 0.05) between the sample and module characteristic genes was used to estimate the correlation between the sample and module.

### 2.5 Validation of DEGs by RT-qPCR

Fifteen key TFs were selected to verify the reliability of the transcriptome data using RT-qPCR. Three biological and three technical replicates were used for RT-qPCR analysis. The Evo M-MLV RT Kit with gDNA Clean for qPCR (Accurate Biotechnology, Changsha, China) was used to synthesize cDNA for RT-qPCR, according to the manufacturer’s instructions. RT-qPCR was performed on a CFX96 Real-Time PCR Detection System (Bio-Rad, USA) using the ChamQ Universal SYBR qPCR Master Mix (Vazyme Biotechnology, Nanjing, China). Specific primers for the 15 selected genes were designed using Primer Premier 5 (USA) software (**Supplementary Table S1**). The 2^−ΔΔCt^ method (Livak and Schmittgen, 2001) was used to calculate relative gene expression, and gene expression levels were normalized to the expression level of the housekeeping gene GAPC2 (Che024479).

### 2.6 Statistical analysis

The visualization and analysis of the data were performed in the bioinformatics platform (https://www.bioinformatics.com.cn) and OriginPro (Version 2021; OriginLab Corporation, Northampton, MA, USA). The fold change correlations between RNA-seq and RT-qPCR were evaluated by the PCC.

## 3 Results

### 3.1 Cytological observation of sex differentiation of flowers treated with exogenous cytokinin

**Figure 1:**
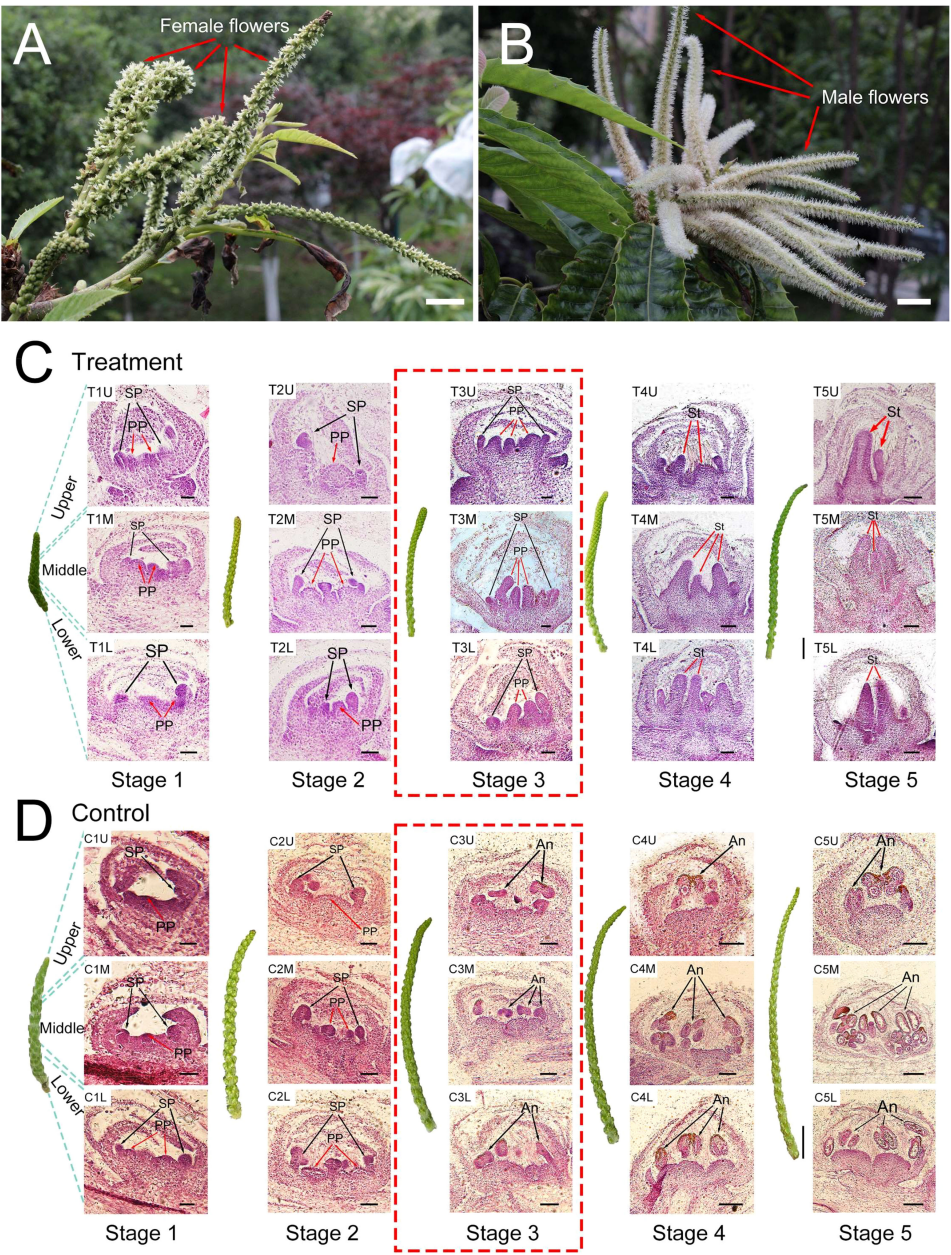
Morphological and cytological effects of exogenous cytokinin on floral development. **(A)** Male inflorescences of *Castanea henryi* treated with exogenous cytokinin (scale bars = 10 mm). **(B)** Male inflorescences of *Castanea henryi* treated with water (scale bars = 10 mm). **(C, D)** Morphological (scale bars = 10 mm) and cytological (scale bars = 200 μm) observation in five stages of the male inflorescences in *Castanea henryi* after exogenous cytokinin or water treatment. The pistil primordium (PP) or stigma (St) is indicated by a red arrow, and the stamen primordium (SP) or anther (An) is indicated by a black arrow.

CK was found to have a considerable feminizing effect on the floral development of *C. henryi*, as demonstrated by the exogenous CPPU treatment. The exogenous CK treatment induced the emergence of female flowers instead of male flowers (**Figures 1A, B**). To investigate the dynamic changes in cell morphology during the feminization of *C. henryi* flowers promoted by exogenous CK, dynamic cytological analysis was performed (**Figures 1C, D**). For simplicity, the focus was on the development of the pistil primordium (PP) and stamen primordium (SP), which directly influence the sex differentiation of flowers.

In the first two stages after CK treatment (stages 1 and 2), the development of PP and SP in the control and treatment groups was at the normal differentiation stage, and there was no significant developmental difference between the two treatments (**Figures 1C, D**). The stasis phenomenon of SP occurred in the third stage after CK treatment; similarly, PP in the control group stagnated in the third stage (**Figures 1C, D**). In contrast, the PP in the treatment group and SP in the control group continued to differentiate and develop rapidly. In the fourth and fifth stages, the PP in the treatment group rapidly elongated and widened, eventually forming a stigma, while the SP in the control group split and grew, eventually forming anthers (**Figures 1C, D**). Hence, the third stage after CK treatment was considered the critical stage of differential differentiation between the pistil and stamen.

### 3.2 Overall transcriptome and sequencing data

**Figure 2:**
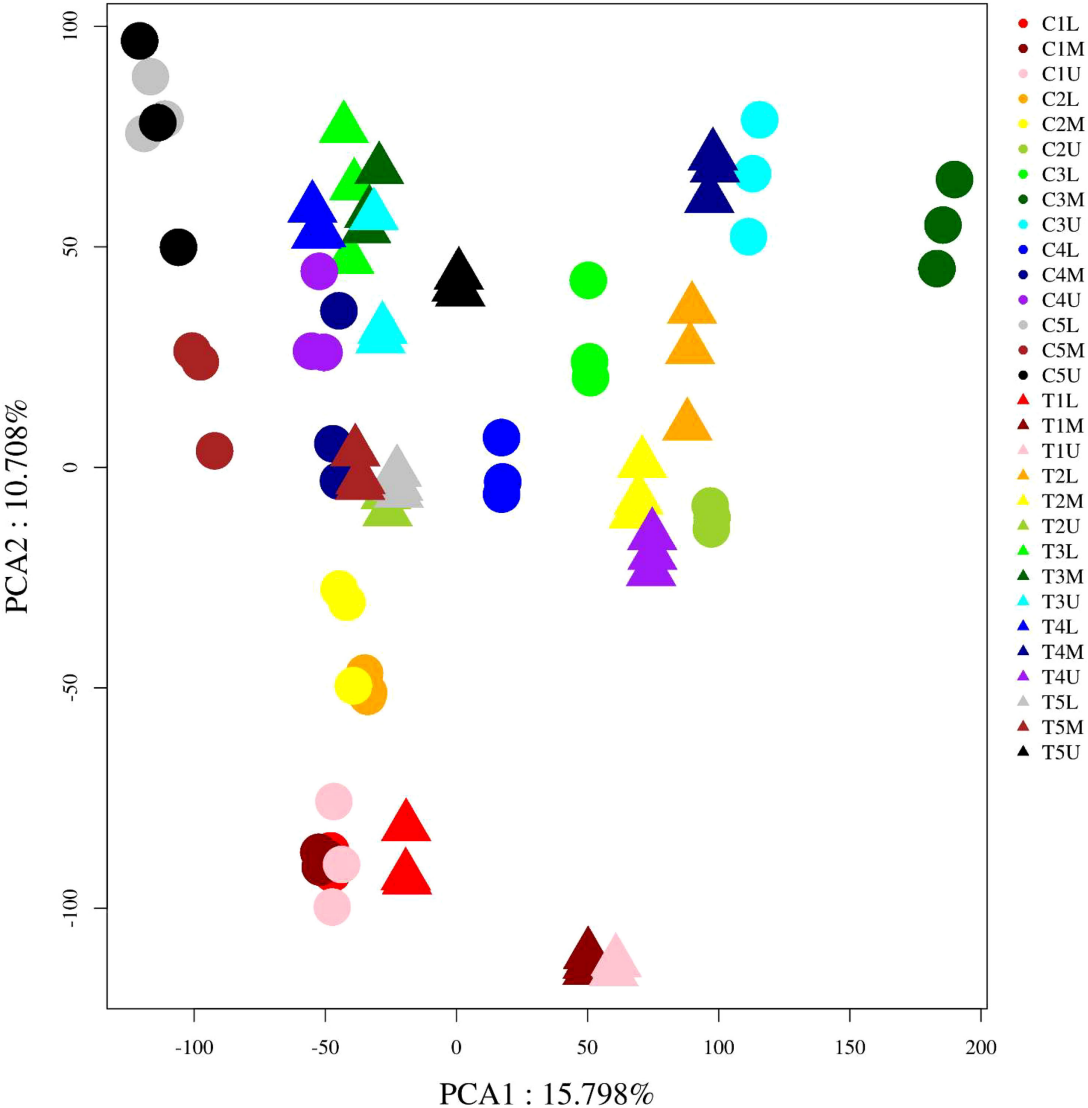
Principal component analysis (PCA) of all RNA-sequence samples. Each point in the PCA score plot indicates an independent biological replicate.

To better explore the molecular mechanism by which CPPU promotes the feminization of male flowers in *C. henryi*, a comparative transcriptome analysis was performed. A total of 90 cDNA libraries were constructed, and high-throughput and quality transcriptome data and 5.26 billion clean reads were obtained through transcriptional data quality control analysis. Each sample produced 36–160 million clean reads, with Q20 and Q30 base percentages greater than 96% and 91%, respectively (**Supplementary Table S2**). The proportion of clean reads that were successfully mapped to the reference genome was greater than 80% (**Supplementary Table S3**). These results indicate that the sequencing data were of reliable quality, and the reference genome was well assembled. The overall gene expression of all samples showed differences in distribution density and dispersion, indicating that different samples had different gene expression levels in response to CPPU treatment (**Supplementary Figure S1**). Two major components of the total variation identified by principal component analysis (PCA), PC1 and PC2, explained 15.8% and 10.7% of the variation in gene expression between samples (**Figure 2**), respectively. However, perhaps because of the short collection time interval between the different samples and the close proximity of the collection sites, the differences between them were not obvious. Sample correlation analysis showed that the samples from similar treatments had a strong correlation, indicating that the identification of different samples was accurate (**Supplementary Figure S2**).

### 3.3 Gene expression in response to exogenous cytokinin treatment

**Figure 3:**
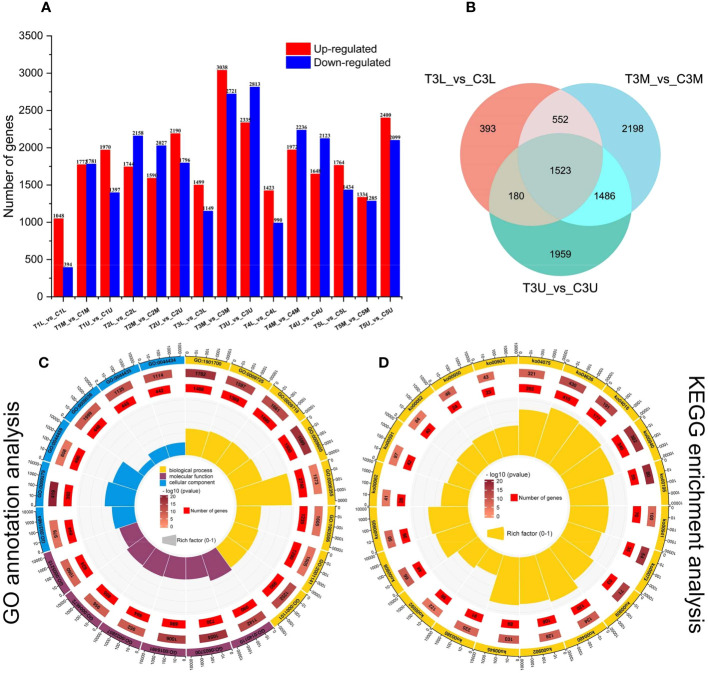
Global transcriptome analysis of three parts in five developmental stages of *C henryi* inflorescences. **(A)** The number of up- (red columns) and down-regulated (blue columns) DEGs in the different comparisons. **(B)** Venn diagram comparison of DEGs in different parts during stage 3. **(C)** GO functional annotation and **(D)** KEGG pathway enrichment analysis of DEGs. The first lap from the outside represents the top 20 GO term IDs or KEGG pathway IDs. The second lap represents the total number of genes included in the specified GO term or KEGG pathway, and the color represents -lg10 P-values for gene enrichment. The third lap represents the number of genes enriched to the specified GO term or KEGG pathway. The fourth lap represents the enrichment factor for each GO term or KEGG pathway. The yellow, blue, and maroon in the GO annotation analysis diagram represent biological processes, molecular functions, and cellular components, respectively.

To investigate the dynamic transcriptional differences between the two treatments at different stages of inflorescence development, we identified genes that were specifically expressed at each stage of inflorescence development in both treatments. For simplicity, we compared pairs of inflorescences of the same site in different treatments to identify DEGs that might be involved in floral sex transition (**Figure 3A** and **Supplementary Table S4**). Among the 15 pair-wise comparisons, T3M_vs_C3M and T3U_vs_C3U showed the largest number of DEGs, reaching a total of 5159 and 5148, respectively. This was consistent with the conclusion of our cytological analysis, which identified the third stage as the critical stage in the differential development of the pistil and stamen primordium (**Figure 3A**). This suggests that the DEGs in the third stage of pair-wise comparison may be a critical candidate gene group for the transition from male to female. A total of 8291 DEGs were identified in the third stage after CPPU treatment, and 1523 genes were differentially expressed in the three parts of the inflorescence (**Figure 3B**).

The DEGs between different samples were further analyzed using GO (**Figure 3C**) and KEGG enrichment analysis (**Figure 3D**). In GO terms, DEGs were significantly enriched (*P* < 0.05) in the following categories: ‘oxygen-containing compound (GO:1901700)’, ‘hormone (GO:0009725)’, ‘endogenous stimulus (GO:0009719)’, ‘external stimulus (GO:0009605)’ and ‘transcription, DNA-templated (GO:0006355)’, which can all be categorized into biological processes (**Figure 3C**). In the KEGG enrichment analysis, the five most enriched metabolic pathways (**Figure 3D**) were plant hormone signal transduction (ko04075), plant-pathogen interaction (ko04626), MAPK signaling pathway (ko04016), phenylpropanoid biosynthesis (ko00940), and photosynthesis (ko00195). These results have important significance for studying the regulatory mechanism by which CPPU promotes floral feminization in *C. henryi*.

### 3.4 Construction of weighted gene co-expression network

**Figure 4:**
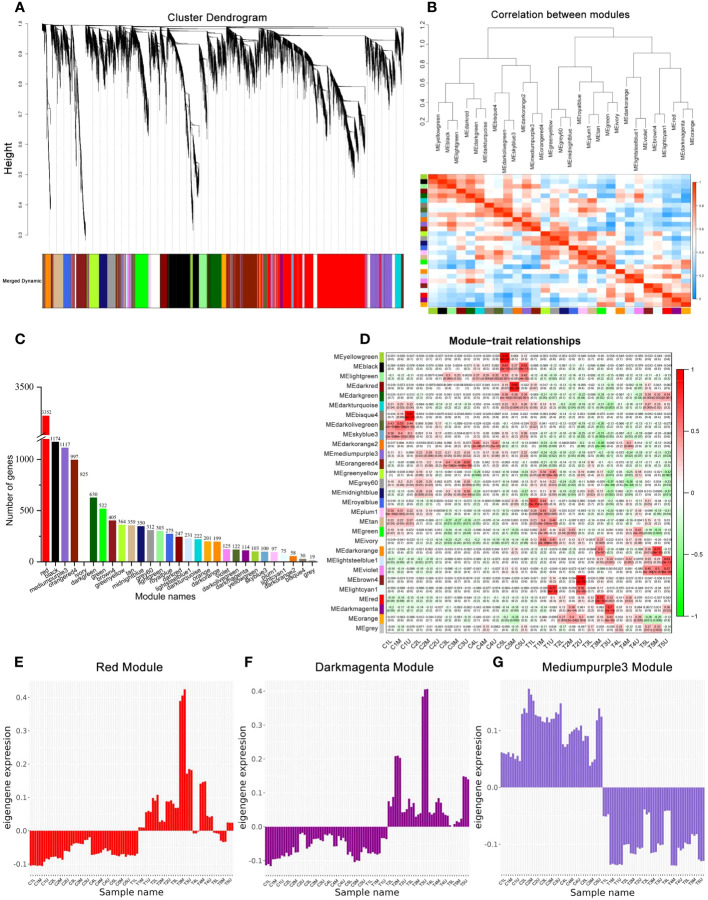
Co-expression network analysis during floral development. **(A)** The modules identified by the weighted gene co-expression network analysis (WGCNA) and hierarchical clustering dendrogram of expressed genes. **(B)** The eigengene network representing the relationships between modules. **(C)** The number of genes in each module. **(D)** Module-sample weight correlations and corresponding P-values. The left panel shows the 29 modules. The colors in the box represent the Pearson correlation coefficient, according to the color legend on the right. **(E–G)** The eigengene expression profiles of the red, darkmagenta, and mediumpurple3 module.

To study the gene regulatory networks of the two treatments during development, weighted gene co-expression network analysis (WGCNA) was used to identify gene co-expression modules. As a result, the cluster dendrogram identified 29 different modules, each of which is represented by a different color (**Figure 4A** and **Supplementary Figure S2**). The correlation between modules was identified using the eigengene dendrogram and heatmap (**Figure 4B**). The number of genes identified in each module ranged from 19 to 3352 (**Figure 4C** and **Supplementary Table S5**), with the red, black, and mediumpurple3 modules each containing more than 1000 genes. Specifically, the red module contained more than 3000 genes (**Figure 4C**).

PCC was used to analyze the correlation between samples in each sample and each module to identify the key modules that may be involved in the sex differentiation of *C. henryi* (**Figure 4D**). Combined with cytological studies (**Figure 1**), we specifically focused on the gene expression of two modules that were significantly correlated with the third stage of flower development. Genes in the red module (3352 genes) were positively correlated with T3M (PCC ≥ 0.7, *P* < 0.05) (**Figure 4D**), while those in the darkmagenta module (114 genes) were positively correlated with T3U (PCC ≥ 0.7, *P* < 0.05) (**Figure 4D**). In these two modules, the expression levels of the eigengenes in the red module were consistent in the control group treated with water and in the first stage after exogenous CK treatment (**Figure 4E**), possibly because the time between the samples in the first stage after treatment and the exogenous CK treatment was too short, resulting in no differences in gene expression being detected. The expression pattern of the eigengenes in another key module (darkmagenta) was also similar to that of the red module (**Figure 4F**). In addition, the expression pattern of the eigengenes in the mediumpurple3 module (1117 genes) opposite between the treatment and control groups (**Figure 4G**), which was consistent with our two treatment patterns, indicating that mediumpurple3 was also a potential key module. These results indicate that these three modular genes are closely involved in the differential development of the pistil and stamen primordium in *C. henryi*.

### 3.5 Functional enrichment analysis of the genes in related modules

**Figure 5:**
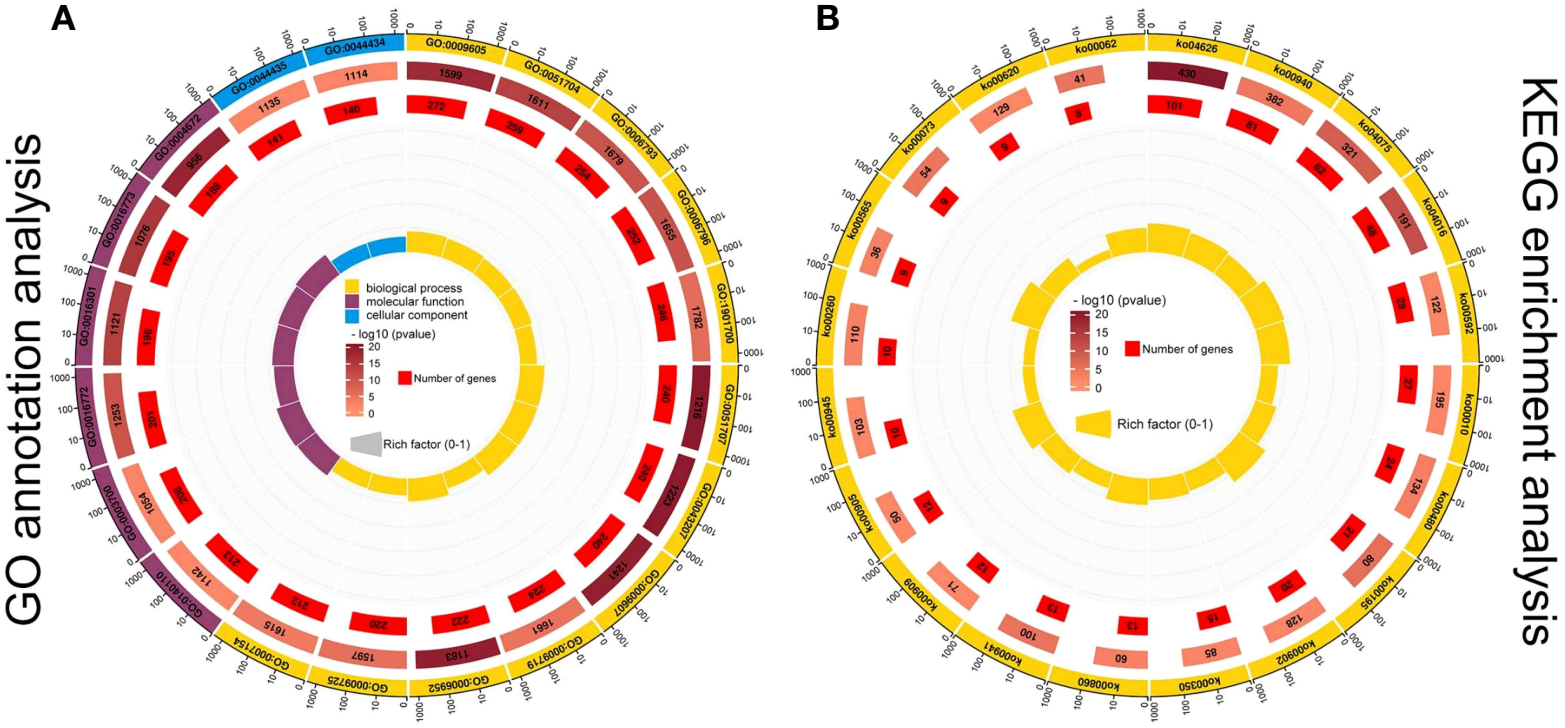
GO functional annotation **(A)** and KEGG pathway **(B)** enrichment analysis of DEGs in three modules. The first lap from the outside represents the top 20 GO term IDs or KEGG pathway IDs. The second lap represents the total number of genes included in the specified GO term or KEGG pathway, and the color represents -lg10 P-values for gene enrichment. The third lap represents the number of genes enriched to the specified GO term or KEGG pathway. The fourth lap represents the enrichment factor for each GO term or KEGG pathway. The yellow, blue, and maroon in the GO annotation analysis diagram represent biological processes, molecular functions, and cellular components, respectively.

To explore the role of DEGs of related modules in sex determination, GO term enrichment (**Figure 5A**) and KEGG (**Figure 5B**) pathway enrichment analyses were conducted to obtain more detailed information about related modules. A total of 647 GO terms were significantly enriched in the red module, 199 GO terms were significantly enriched in the dark magenta module, and 293 GO terms were significantly enriched in the mediumpurple3 module (*P* < 0.05). Most of the DEGs in the red, darkmagenta, and mediumpurple3 modules were enriched in the biological process category, and most of them were classified as external stimuli (GO:0009605), multi-organism process (GO:0051704), phosphorus metabolic process (GO:0006793), phosphate-containing compound metabolic process (GO:0006796), or oxygen-containing compound (GO:1901700) (**Figure 5A**). A large number of DEGs were enriched in transcription regulator activity (GO:0140110), DNA-binding transcription factor activity (GO:0003700), transferase activity, transferring phosphorus-containing groups (GO:0016772), and kinase activity (GO:0016301), which can all be categorized as molecular functions (**Figure 5A**). In addition, a few DEGs were enriched in the cellular component category of the plastid part (GO:0044435) or chloroplast part (GO:0044434).

Similarly, the DEGs in the three modules were enriched in the KEGG pathway to reveal their potential role in sex differentiation of *C. henryi* (**Figure 5B**). Among the related modules, DEGs were mainly enriched in the plant-pathogen interaction (ko04626), phenylpropanoid biosynthesis (ko00940), plant hormone signal transduction (ko04075), MAPK signaling pathway (ko04016), and alpha-linolenic acid metabolism (ko00592) pathways (**Figure 5B**). Taken together, these results provide a transcriptional overview to understand the functional enrichment classification of key modules.

### 3.6 Differential expression of cytokinin biosynthesis and signaling genes in related modules

**Figure 6:**
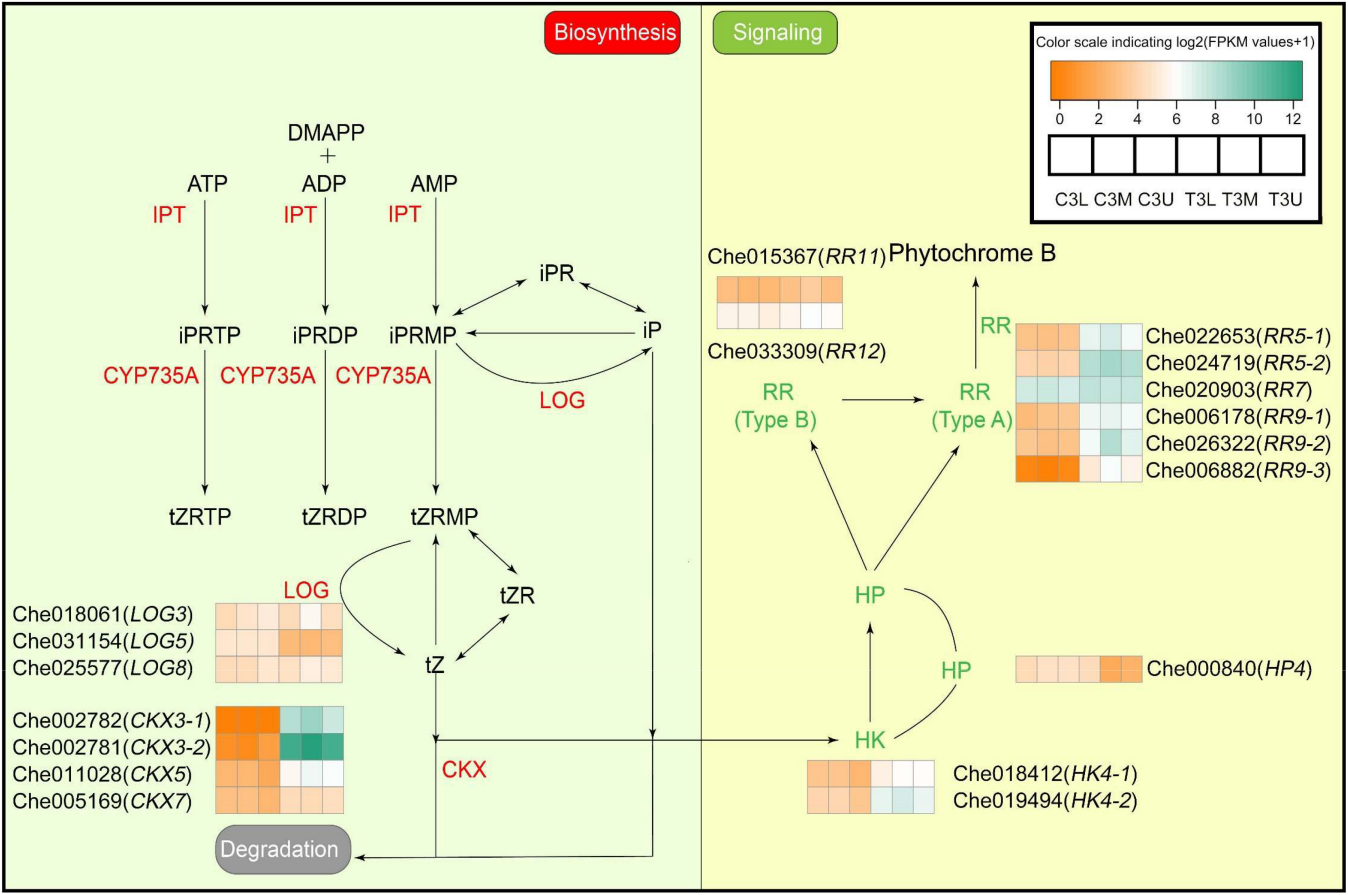
Differential expression of cytokinin biosynthesis and signaling genes in related modules at different parts in stage 3. The color scale indicates log2 (Fragments per kilobase of exon per million reads mapped values + 1). Different colors represent the level of gene expression according to the color scale. DMAPP, Dimethylallyl diphosphate; ATP, Adenosine triphosphate; ADP, Adenosine diphosphate; AMP, Adenosine monophosphate; IPT, isopentenyltransferase; iP, N6-isopentenyladenine; iPR, isopentenyladenosine; IPRTP, iP riboside 5’-triphosphate; iPRDP, iP riboside 5’-diphosphate; iPRMP, iP riboside 5’-monophosphate; CYP735A, cytochrome P450 monooxygenase, family 735, subfamily A; tZ, trans-zeatin; tZR, trans-zeatin riboside; tZRTP, tZ riboside 5’-triphosphate; tZRDP, tZ riboside 5’-diphosphate; tZRMP, tZ riboside 5’-monophosphate. Other abbreviations are as presented in the text.

The response of endo- to exogenous CK was studied by identifying the genes related to endogenous CK biosynthesis and signaling. A total of seven DEGs involved in CK biosynthesis and metabolism (**Figure 6** and **Supplementary Table S6**), including three *cytokinin riboside 5’-monophosphate phosphoribohydrolases*, *LOG3* (Che018061), *LOG5* (Che031154), and *LOG8* (Che025577), and four *cytokinin dehydrogenases*, *CKX3* (Che002782 and Che002781), *CKX5* (Che011028), and *CKX7* (Che005169), were identified in three related modules. In addition, the expression patterns of the two gene families (*LOGs* and *CKXs*) were the opposite in the control and treatment groups. The expression of *LOGs*, one of the main synthetases of CK, was significantly downregulated after treatment with exogenous CK, whereas the expression of *CKXs*, the only degrading enzyme of CK, was significantly upregulated. It is worth noting that the FPKM values of *CKX3-1* (Che002782) and *CKX3-2* (Che002781) in the control group were approximately 0 and 0.5, respectively, whereas those of these two genes in the corresponding exogenous CK treatment group reached approximately 300 and 2,500, respectively (**Figure 6**). This indicates that exogenous CK treatment greatly promoted the expression of cytokinin dehydrogenases and the degradation of endogenous CK, and greatly changed the homeostasis of endogenous CK.

Furthermore, genes involved in the CK signaling pathway were significantly differentially expressed after treatment with exogenous CK. Eleven DEGs involved in CK signaling pathways were identified in the key modules (**Figure 6** and **Supplementary Table S6**). The expression levels of two histidine kinases, *HK4* (Che018412 and Che019494), a *histidine-containing phosphotransfer protein*, *HP4* (Che000840), six type-A *two-component response regulators*, *RR5* (Che022653 and Che024719), *RR7* (Che020903), *RR9* (Che006178, Che026322, and Che006882), and two type-B *two-component response regulators*, *RR11* (Che015367) and *RR12* (Che033309), responded to exogenous CK treatment. Notably, the expression levels of six type-A *RRs* and two *HKs* were significantly upregulated under exogenous CK treatment (**Figure 6**), which suggests that the two have a strong feedback regulation of exogenous CK treatment, and the transcription of downstream target genes of CK could be promoted by exogenous CK treatment.

### 3.7 Differential expression of gibberellin metabolism and signaling genes in related modules

**Figure 7:**
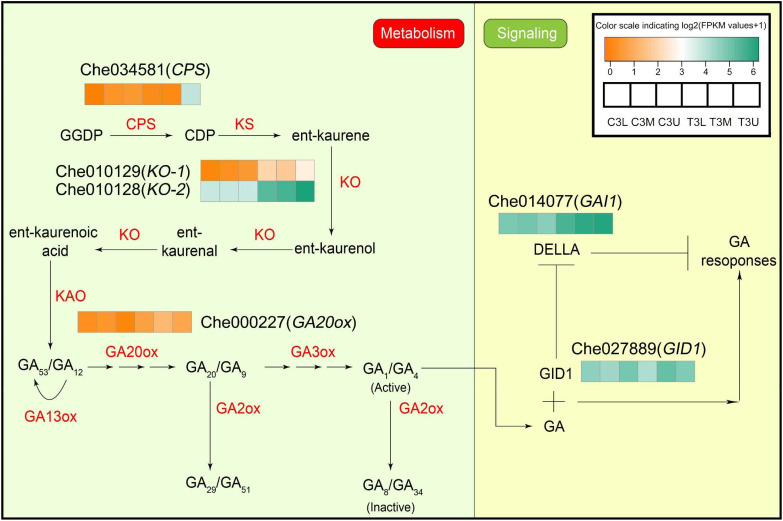
Differential expression of gibberellin metabolism and signaling genes in related modules at different parts in stage 3. The color scale indicates log2 (Fragments per kilobase of exon per million reads mapped values + 1). Different colors represent the level of gene expression according to the color scale. GGDP, trans-geranylgeranyl diphosphate; CDP, ent-copalyl diphosphate. Other abbreviations are as presented in the text.

In contrast to the endogenous CK response to CPPU treatment, only six DEGs were identified in the GA biosynthesis and signaling pathway (**Figure 7** and **Supplementary Table S7**). In the GA biosynthesis pathway, *copalyl diphosphate synthase* (*CPS*, Che034581), two *ent-kaurene oxidases* (*KO*, Che010129 and Che010128), and *gibberellin 20-oxidase-like protein* (*GA20ox*, Che000227) were significantly differentially expressed in response to CPPU treatment. In particular, the expression levels of GA synthetase, *KO-1*, and *KO-2* were higher in the treated group than in the control group, indicating that ent-kaurenol synthesis was higher in the treated group than in the control. Moreover, two GA signaling-related genes, *gibberellin receptor GID1B-like* (Che027889) and *DELLA protein GAI1* (Che014077), were also regulated by exogenous CK treatment (**Figure 7**). The expression levels of both genes were significantly upregulated in response to the exogenous CK treatment. This indicates that endogenous GA responded significantly to exogenous CK treatment.

### 3.8 Response of genes related to auxin and ABA biosynthesis and signaling to cytokinin treatment

**Figure 8:**
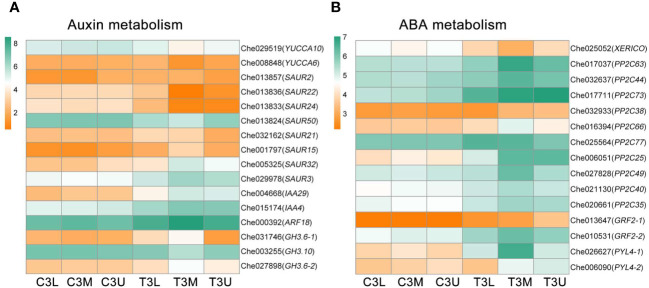
Differential expression of auxin and abscisic acid metabolism genes in related modules at different parts in stage 3. The color scale indicates log2 (Fragments per kilobase of exon per million reads mapped values + 1). **(A)** The gene expression profiles of auxin metabolism pathway in related modules at different parts in stage 3. **(B)** The gene expression profiles of abscisic acid metabolism pathway in related modules at different parts in stage 3.

Differential expression of numerous genes involved in auxin biosynthesis and signaling was observed under cytokinin treatment, as indicated by gene annotation results. Specifically, the expression levels of two auxin biosynthesis-related genes, *YUCCA6* (Che008848) and *YUCCA10* (Che029519), were significantly downregulated, while 14 genes associated with auxin signaling exhibited differential expression following cytokinin treatment (**Figure 8A**). Among them, *YUCCA*, encoding crucial flavin monooxygenases involved in auxin biosynthesis, showed decreased expression levels after cytokinin treatment, suggesting a potential transient reduction in endogenous auxin levels at stage 3. Additionally, cytokinin treatment regulated the expression of 15 genes involved in abscisic acid (ABA) biosynthesis and signaling (**Figure 8B**). The ABA biosynthesis-related gene *XERICO* (Che025052) was significantly downregulated after CPPU treatment, while cytokinin treatment positively influenced the expression of ABA signaling-related genes, indicating a strong feedback regulatory role of cytokinin in ABA signaling. These discoveries provide critical insights into the intricate relationship between auxin and ABA in sex determination of *C. henryi* under cytokinin regulation.

### 3.9 Identification of transcription factors and flowering−associated genes for related modules

**Figure 9:**
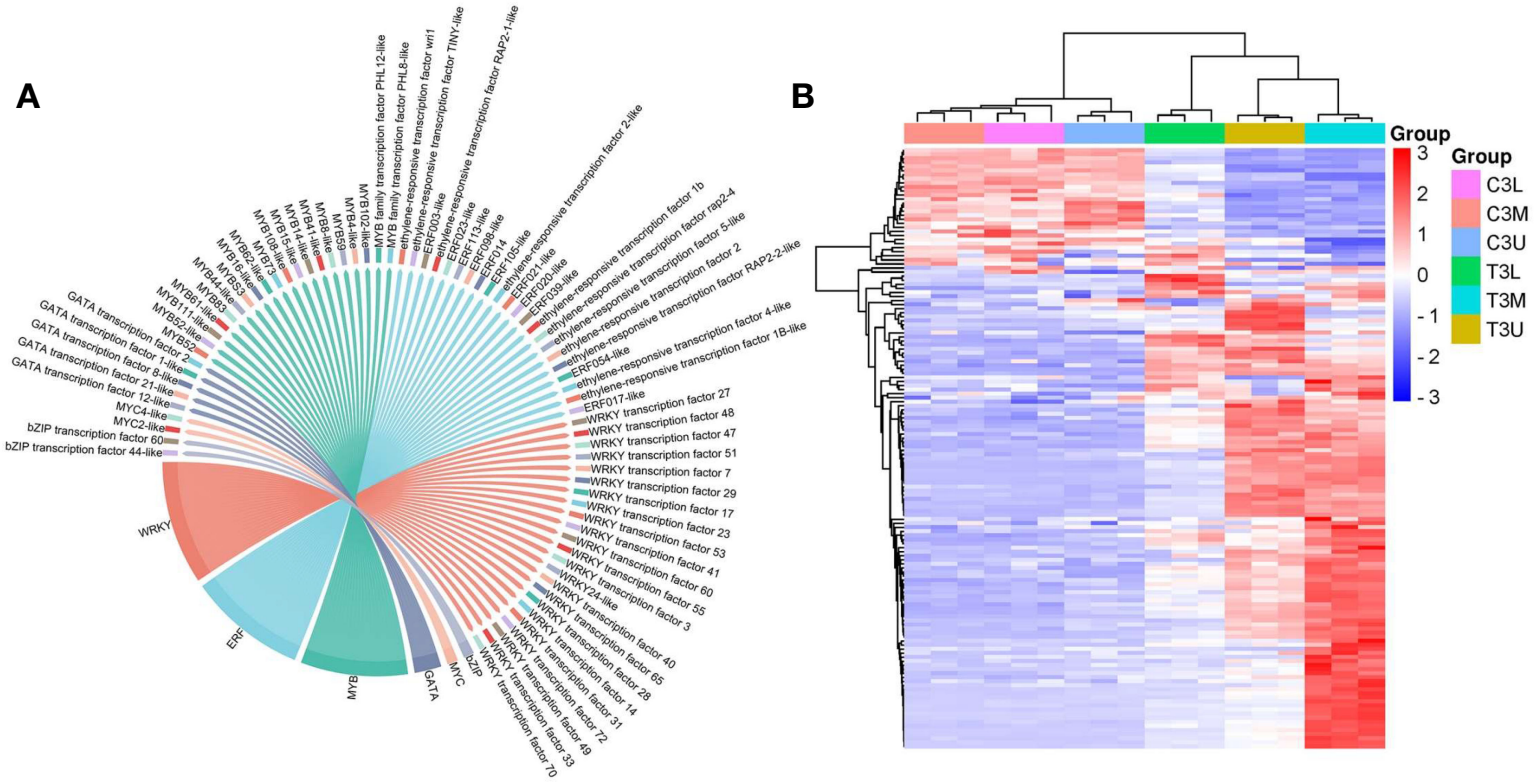
Transcription factor family statistics and heatmap analysis in the red, darkmagenta, and mediumpurple3 modules. **(A)** The top 6 most abundant transcription factor families in three modules. **(B)** Hierarchical clustering of the TFs in three modules at different parts in stage 3.

Identification of TFs involved in important biological processes is crucial for elucidating the underlying regulatory mechanisms. In total, 148 expressed TFs belonging to 71 TF families were identified in these three modules. The six most abundant TF families were *WRKY* (23), *ERF* (22), *MYB* (20), *GATA* (5), *MYC* (2), and *bZIP* (2) (**Figure 9A**). There were 74 TFs in these families, representing half of the total number of identified TFs. Additionally, the expression levels of most identified TFs were significantly upregulated after exogenous CK treatment (**Figure 9B**), indicating that most TFs responded to exogenous CK treatment by increasing their transcriptional activities.

TFs with the top 15 expression differences were considered potential critical regulators of the related modules (**Table 1**). The expression patterns of the top 15 TFs were upregulated after CPPU treatment, which mainly included *WRKY* (8) and *ERF* (5) family members. These genes may potentially regulate floral feminization in *C. henryi*, which warrants further study. In particular, the log2FC values of *WRKY transcription factor 40/47/51*, *ERF021-like*, and *MYB4-like* exceeded 5, indicating that these genes were highly sensitive and responsive to exogenous CK treatment.

**Table 1:** The expression patterns of the top 15 TFs (displayed with a white background) with different expression levels and 11 DEGs related to the development of flower organs (displayed with an off-white background) at different parts in stage 3.

Gene ID	Nr annotation	log2 Fold Change		
		T3L_vs_C3L	T3M_vs_C3M	T3U_vs_C3U
Che015211	WRKY transcription factor 47	2.06	6.16	3.56
Che002053	WRKY transcription factor 40	1.76	5.12	2.13
Che002052	WRKY transcription factor 60	1.67	4.48	2.66
Che002539	WRKY transcription factor 51	1.66	5.07	2.03
Che035845	WRKY transcription factor 53	1.74	3.16	3.37
Che003944	WRKY transcription factor 41	1.63	3.08	3.16
Che018581	WRKY transcription factor 33	1.46	3.1	3.01
Che020428	WRKY transcription factor 41	1.47	3.27	2.83
Che031586	ERF021-like	2.8	6.01	3.41
Che017366	ERF098-like	1.64	4.03	1.84
Che024313	ERF1b	0.91	4.58	1.97
Che002017	ERF003-like	2.83	2.55	3.03
Che031597	ERF020-like	1.62	3.57	1.83
Che031520	MYB4-like	3.41	6.24	2.81
Che017379	MYB4-like	2.1	3.85	2.64
Che002541	agamous-like MADS-box protein AGL11	5.29	7.6	4.04
Che010997	floral homeotic protein DEFICIENS	-1.49	-2.05	-3.44
Che017550	agamous-like MADS-box protein agl15	-1.57	-1.81	-3.02
Che025647	developmental protein SEPALLATA 1-like	1.11	2.89	-1.83
Che009216	mads-box protein svp	0.84	0.41	0.49
Che005357	agamous-like MADS-box protein AGL9 homolog	-0.22	-0.85	-1.39
Che002592	AGL2-like MADS box 3	-0.44	-0.24	-1.68
Che012257	MADS-box protein JOINTLESS	1.01	1.88	1.41
Che007074	MADS-box transcription factor 17-like	-0.45	0.57	2.25
Che020918	floral homeotic protein apetala 2	-0.98	-1.87	-1.42
Che012336	UNUSUAL FLORAL ORGANS-like	-0.5	-1.93	-1.51

The dark-to-light green color indicates a down-regulation fold of genes from high to low, while the light-to-dark red color indicates an up-regulated fold of genes from low to high.

Furthermore, 11 DEGs related to the development of flower organs in the module were identified (**Table 1**). Notably, the D-class MADS-box *agamous-like MADS-box protein AGL11* (Che002541) showed the greatest difference in expression between the two treatments, reaching a log2FC value of 7.6, followed by the B-class Mads-box gene *floral homeotic protein DEFICIENS* (*DEF*, Che010997), MADS-box gene *agamous-like MADS-box protein AGL15* (Che017550), and the developmental protein *SEPALLATA 1-like* (*SEP1*, Che025647), which significantly changed the expression levels in response to exogenous CK treatment. In conclusion, the identification of CK-sensitive DEGs associated with floral organ development provides insights into flower sex determination in *C. henryi*.

### 3.10 Validation of expression profiles of candidate genes by RT-qPCR

**Figure 10:**
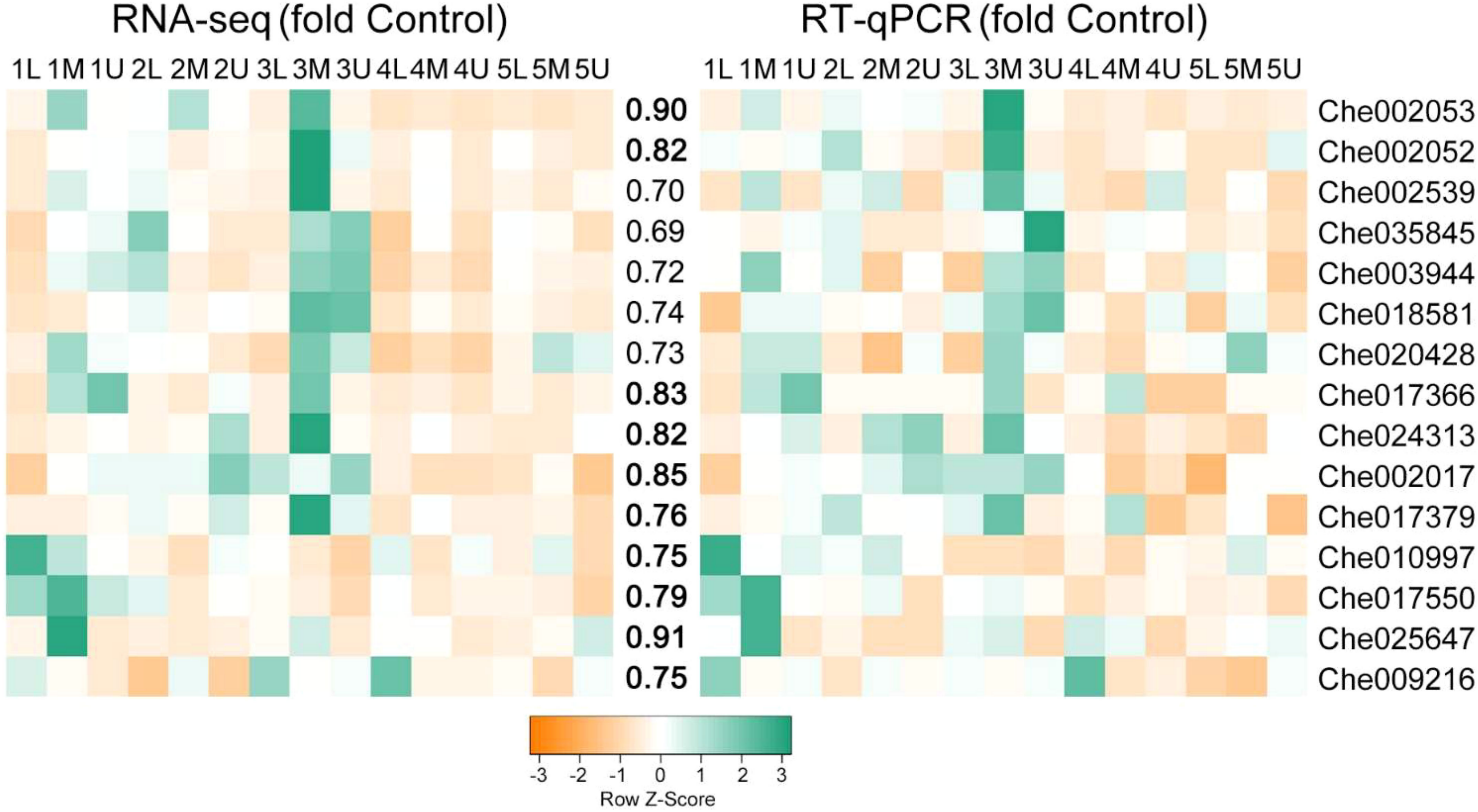
Correlation between expression profiles of RNA-seq and RT-qPCR of selected genes. Heatmaps represent the expression profiles of selected genes (markers on the right) from RNA-seq (left) and RT-qPCR (right) analyses. The value between the two heatmaps represents the correlation value between the expression profiles analyzed by RNA-seq and RT-qPCR for each gene, with the correlation values above 0.75 highlighted in bold.

To verify the reliability of the transcriptome sequencing data, the 15 key TFs identified in this study were selected for RT-qPCR validation. The expression patterns of the RT-qPCR results for these genes were consistent with those of the FPKM values obtained by RNA-Seq, and were significantly correlated (*P* < 0.01) (**Figure 10**). The correlation coefficient between RNA-seq and RT-qPCR analysis for most of the tested genes was ≥ 0.75 (**Figure 10**), indicating that the accuracy of the RNA-seq data reflected the abundance of transcript levels.

## 4 Discussion

### 4.1 Exogenous cytokinin treatment promotes the conversion of male to female flowers

Sex differentiation in plants is a special type of organogenesis, that is, the formation of stamens or pistils, which includes female and male sex determination and gametophyte differentiation, development, and maturation (Durand and Durand, 1984). Unisexuality in plants is usually caused by the reduction or abortion of sex organ primordia. In the early stages of unisexual flower development, two sex organ primordia appear simultaneously, which is called the ‘bisexual stage’. Under the action of sex determination genes, one of the primordia is arrested at a particular stage, resulting in the abortion and loss of function of that particular reproductive organ, whereas the opposite reproductive organs develop normally to sexual maturity (Dellaporta and Calderon-Urrea, 1993).

Plant sex is generally determined at a certain stage after growth, differentiation, and development. Therefore, plant sex differentiation is easily affected by external environmental conditions, such as phytohormones, temperature, nutrition, and other factors (Malepszy and Niemirowicz-Szczytt, 1991). In the normal differentiation process, male flowers of *C. henryi* have a ‘bisexual stage’, where the female and stamen primordium coexist. After selective induction or abortion of the sex organs, the development of the pistil primordium stops, stamens continue to extend, and the unisexual male flowers ultimately form.

In the present study, the mechanism of sex differentiation in *C. henryi* was reversed by exogenous CK treatment. After the ‘bisexual stage’ of the bisexual primordium coexistence, exogenous CK induced the development of the pistil primordium and inhibited the elongation of the stamen primordium, which ultimately led to the conversion of male flowers to female. The ability to promote feminization of male *C. henryi* flowers by CK treatment suggests that even pistil primordium, which would normally be aborted, can potentially develop into a complete pistil under induction by exogenous CK.

### 4.2 Cytokinin and gibberellin biosynthesis and signaling-related genes likely contribute to flower sex transition

In the CK biosynthesis pathway in plants, the precursor substances of CK are converted into physiologically active CK, including a two-step activation pathway and a direct activation pathway. The former involves the conversion of CK nucleotides to their active forms in a two-step reaction catalyzed by nucleotidase (Chen and Kristopeit, 1981a) and nucleosidase (Chen and Kristopeit, 1981b). However, to date, no corresponding enzyme specifically involved in this reaction pathway has been identified. The direct activation pathway can directly transform precursor substances into biologically active CK through enzymes of the *LONELY GUY* (*LOG*) family (Kuroha et al., 2009). The gene *LOG* was isolated from rice for the first time and could directly convert iPRMP and tZRMP into iP and tZ. In rice, deletion of this gene leads to rapid termination of the inflorescence meristem and branch meristem after the production of a small amount of lateral meristem, accompanied by abnormal floral organ development. Most flowers have only one stamen and no pistils, ultimately leading to decreased rice yield (Kurakawa et al., 2007).

In contrast, *CKX* is the only known enzyme involved in the degradation of CK (Schmülling et al., 2003). Overexpression of *ZmCKX1* in maize and tobacco resulted in male sterility and affected the normal function of stamens (Galuszka et al., 2005). In this study, exogenous CK treatment induced strong upregulation of the *CKX* gene family expression and strong downregulation of the *LOG* gene family expression. Interestingly, as a member of the A-class MADS-box family with functions that regulate floral meristem and floral organ morphology (Gustafson-Brown et al., 1994), *APETALA 1* (*AP1*) can regulate CK levels by directly suppressing the CK biosynthesis gene *LOG1* and activating the CK degradation gene *CKX3* to suppress meristem activity in sepal axils (Han et al., 2014). This regulatory mechanism directly linked genes that clearly affect flower organ development to CK homeostasis regulation, possibly suggesting that the CK biosynthesis-related genes in our study were involved in sex transition in *C. henryi*. Thus, it is possible to control floral organ regeneration by regulating the expression of the genes involved in CK biosynthesis.

In addition, in exogenous CK-induced carpel regeneration in *Arabidopsis*, the CK signaling type-B *ARABIDOPSIS RESPONSE REGULATOR* (*ARR*)*1* and *ARR10* directly bind to the *AGAMOUS* (*AG*) promoter, thereby inducing the expression of the carpel identity gene (Rong et al., 2018). Thus, type-B ARRs can control carpel regeneration by mediating *AG* expression. Moreover, the phytohormone signaling pathway is considered a complex network (Cui and Luan, 2012). To regulate plant growth and development, CK interacts with various phytohormones, such as auxin (Moubayidin et al., 2009), ethylene (Zdarska et al., 2015), abscisic acid (Tran et al., 2007), GA (Greenboim-Wainberg et al., 2005), and jasmonic acid (Ueda and Kato, 1982). Therefore, the interaction of the CK signaling pathway with other phytohormones should not be ignored in floral sex determination.

Genes encoding the initial steps of GA biosynthesis were highly expressed in the exogenous CK-treated group in this study. For ferns, an antheridiogen-mediated communication system has evolved to produce males by modifying the GA biosynthetic pathway, which is split between two individuals at different developmental stages in the colony (Tanaka et al., 2014). Similarly, antheridiogens are produced in the developing prothallia of various ferns, all of which are GA-related compounds (Yamane, 1998), suggesting that the GA biosynthetic pathway may be used to at least partly synthesize androgens in ferns.

The GID1 protein is a soluble GA receptor that can bind to active GA, sense and transmit GA signals, and thus induce a series of downstream reactions (Ueguchi-Tanaka et al., 2005). GA derepresses the GA signaling pathway by GID1-induced degradation of DELLA proteins. As a growth suppressor in the GA signaling pathway, the DELLA protein delays flowering and controls floral transition by inhibiting the floral meristem-identity genes *LEAFY* (*LFY*) and *SUPPRESSOR OF OVEREXPRESSION OF CONSTANS 1* (*SOC1*) (Achard et al., 2007). GID1 and DELLA proteins were identified in this study, suggesting that GA signaling pathways may be involved in flower transition.

### 4.3 Transcription factors may participate in floral sex determination

TFs are a class of proteins that bind to specific DNA-regulatory sequences (enhancers and silencers) and are generally localized in the upstream region of target genes to modulate the rate of gene transcription (Meshi and Iwabuchi, 1995). In this study, the expression levels of TFs, including WRKYs, ERFs, and MYBs, were significantly differentially expressed under the exogenous CK treatment. TFs are involved in regulating various processes of plant growth and development (Katagiri and Chua, 1992), therefore, the study of the three families of TFs is conducive to a better understanding of sex determination in *C. henryi*.

The *WRKY* TF family, whose most extensive function in plants is part of a complex hormone signaling network, acts upstream and downstream of hormones and participates in the plant’s response to environmental stress (Bakshi and Oelmüller, 2014). The WRKY34 TF, a male gametophyte-specific group I WRKY family member (Lei et al., 2017), is involved in pollen development and is regulated by the pollen-specific MIKC class of MADS-domain TFs under cold stress (Zou et al., 2010). This indicates that the WRKY TF family is involved in the later development of male gametophytes.

The *ERF*/*APETALA2* (*AP2*) TF family plays a central role in the establishment of the floral meristem, specification of floral organ identity, and regulation of floral homeotic gene expression in *Arabidopsis* (Jofuku et al., 1994). An important function of *ERF*/*AP2* is to regulate flower organ development. In strong *ERF/AP2* mutants of *Arabidopsis*, sepals transform into carpel-containing ovules, petal development is suppressed, and stamen numbers are reduced (Komaki et al., 1988; Kunst et al., 1989), possibly indicating the upregulated expression of *ERF*/*AP2* TF affected the normal development of stamens.

*MYB* are a large family of TFs that are widely distributed in plants and are involved in the regulation of a large number of genes related to floral organ differentiation. In *Arabidopsis thaliana*, downregulation of the TF *AtMYB103* using transgenic technology results in early tapetal degeneration and pollen aberration during another development. This would cause retention of the nutrients required for microspore development, thus making suppressing normal pollen development (Zhang et al., 2007); inhibiting its expression would result in complete male sterility (Phan et al., 2012).

### 4.4 A regulatory network for cytokinin in promoting floral feminization

As *C. henryi* flowers contain only one type of functional sex organ, unisexual flowers are an ideal model for further study of the regulatory mechanism of plant sex determination (Aryal and Ming, 2014). Here, we successfully transformed male *C. henryi* flowers into female flowers by applying exogenous CK to male inflorescences. Therefore, it is of great interest to study the molecular mechanism of male flower feminization to understand sex determination in *C. henryi*. The present study indicated that genes related to CK and GA biosynthesis and signaling pathways responded positively to exogenous CK treatment. Similarly, several TFs that indirectly affect flower organs were identified, including *WRKY47*, *ERF021*, and *MYB4*, with large differences. In addition, the responses of flower organ recognition genes *AGL11*/*15*, *DEF*, and *SEP1* (for more detailed information on the genes involved, see also ref. (Shore and Sharrocks, 1995; Pelaz et al., 2000; Zahn et al., 2005; Adamczyk et al., 2007; Huang et al., 2017)) to exogenous CK treatment is considered a critical factor in flower organ sex transition.

**Figure 11:**
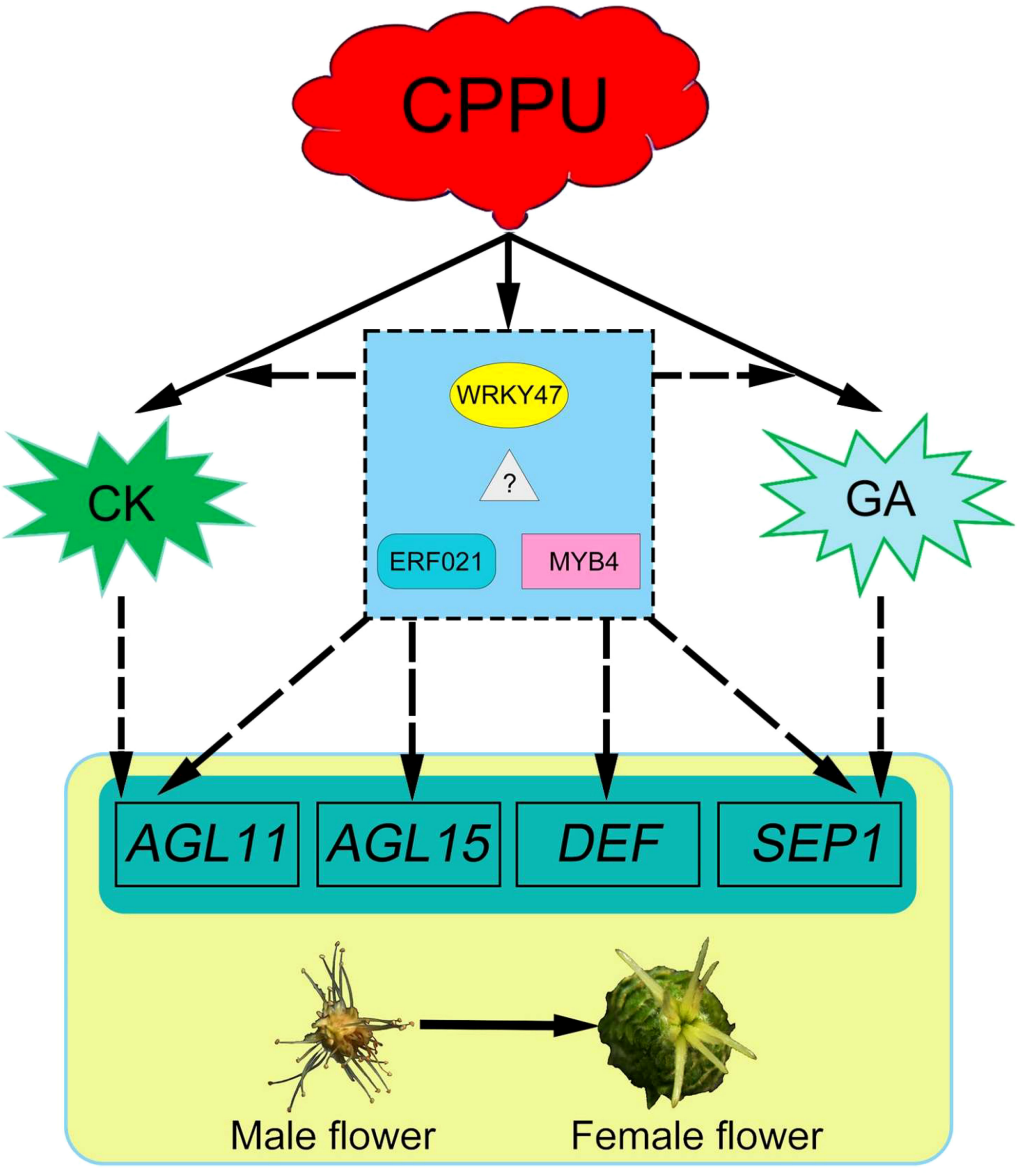
A conceptual model for cytokinin in promoting floral feminization in *Castanea henryi*. The dashed line represents the hypothetical regulatory relationship.

Collectively, exogenous CK can directly or indirectly regulate the expression of genes involved in CK and GA biosynthesis and signaling. TFs, including *WRKY47*, *ERF021*, and *MYB4*, were also found to participate in CK-mediated floral transition. Eventually, the significant differential expression of several genes involved in flower organ recognition led to the transition from androecium to gynoecium in *C. henryi* (**Figure 11**). However, the key genes identified in this study were screened using transcriptomic data. Understanding the function of these genes in *C. henryi* in more detail requires further investigation.

## 5 Conclusions

In the present study, exogenous CK induced the transformation of male flowers into female flowers in *C. henryi*. Based on dynamic cytological and transcriptomic analyses, we constructed a co-expression regulatory network using WGCNA for five stages after CK treatment and identified CK and GA biosynthesis genes supplemented with signaling-related genes and TFs that responded positively to exogenous CK treatment in key modules. Based on identified candidate genes, we proposed a genetic regulatory network of exogenous CK for flower sex determination in *C. henryi*, which provides target genes for future studies. These findings provide new insights into the molecular mechanisms of hormonal regulation of sex determination in *C. henryi*, and present reference information for improving the fruit yield of *C. henryi*.

## Data availability statement

The datasets presented in this study can be found in online repositories. The names of the repository/repositories and accession number(s) can be found in the article/**Supplementary Material**.

## Author contributions

GW: Data curation, Formal Analysis, Investigation, Methodology, Software, Validation, Visualization, Writing – original draft, Writing – review & editing. XT: Conceptualization, Data curation, Formal Analysis, Methodology, Software, Validation, Writing – review & editing. QQ: Investigation, Methodology, Software, Writing – review & editing. YZ: Formal Analysis, Validation, Visualization, Writing – review & editing. XF: Conceptualization, Funding acquisition, Methodology, Supervision, Writing – review & editing. DY: Funding acquisition, Project administration, Resources, Supervision, Writing – review & editing.

## Supplementary material

Supplementary Figure 1 The boxplot of Fragments per kilobase of exon per million reads mapped (FPKM) of all samples.

Supplementary Figure 2 The correlation between samples, as indicated by the Pearson correlation coefficient of gene expression for each sample.

Supplementary Figure 3 A clustering dendrogram of genes with dissimilarity based on the topological overlap, as well as the specified module colors.

Supplementary Table 1 Primers used for real-time quantitative polymerase chain reaction (RT-qPCR).

Supplementary Table 2 The statistics of reads before and after filtering.

Supplementary Table 3 The statistical results of clean reads aligned to the reference genome.

Supplementary Table 4 The number of up- and down-regulated differentially expressed genes (DEGs) in different comparisons.

Supplementary Table 5 The modules and members in modules identified by weighted gene co-expression network analysis (WGCNA).

Supplementary Table 6 The FPKM values of cytokinin biosynthesis and signaling genes in related modules at stage 3.

Supplementary Table 7 The FPKM values of gibberellin metabolism and signaling genes in related modules at stage 3.
