# Supplementary figures and images for: Dynamic cytological and transcriptomic analyses provide novel insights into the mechanisms of sex determination in Castanea henryi
Source: Front Plant Sci. 2023 Sep 12;14:1257541. doi: 10.3389/fpls.2023.1257541 (PMC10523332; doi:10.3389/fpls.2023.1257541)

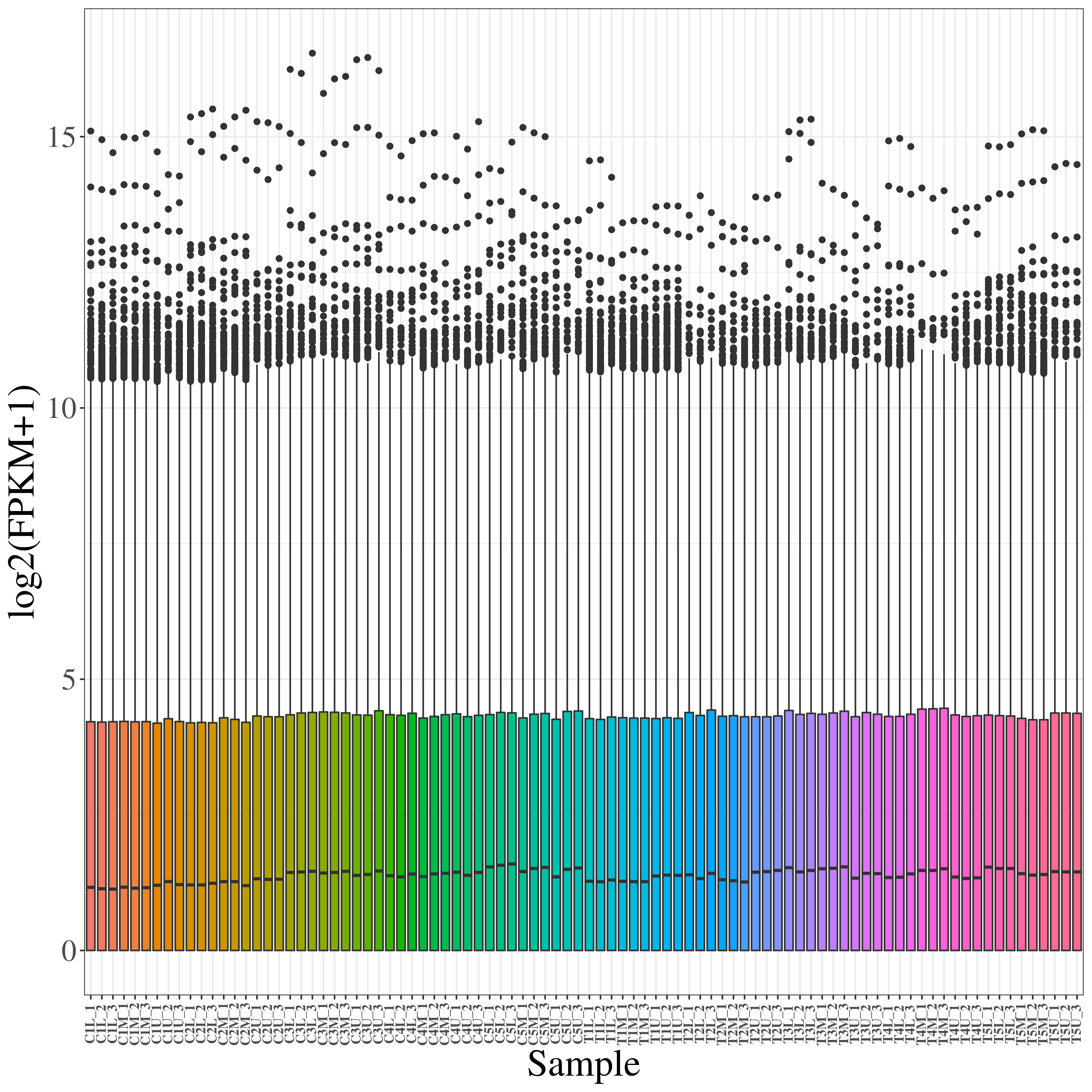

Supplement: Supplementary Figure 1 — The boxplot of Fragments per kilobase of exon per million reads mapped (FPKM) of all samples. [file DataSheet_1.zip › Supplementary Figure S1.jpg]

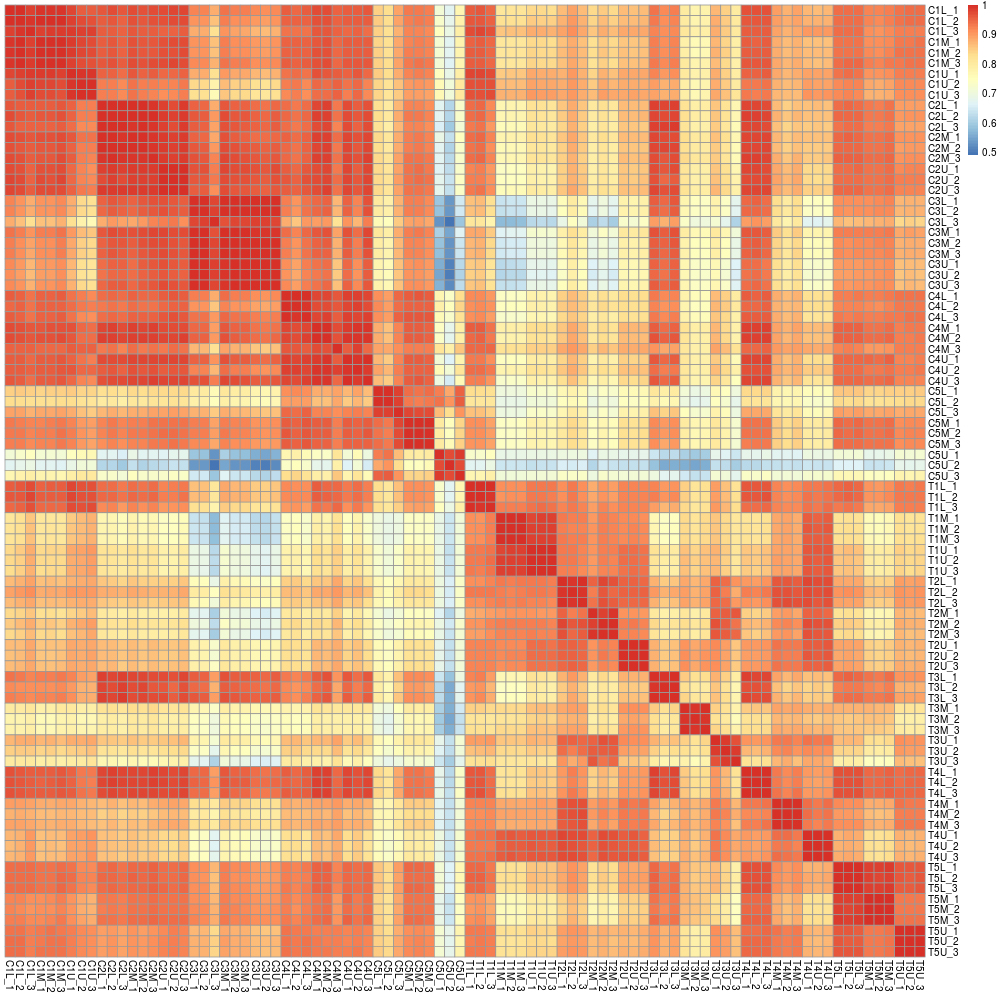

Supplement: Supplementary Figure 1 — The boxplot of Fragments per kilobase of exon per million reads mapped (FPKM) of all samples. [file DataSheet_1.zip › Supplementary Figure S2.jpg]

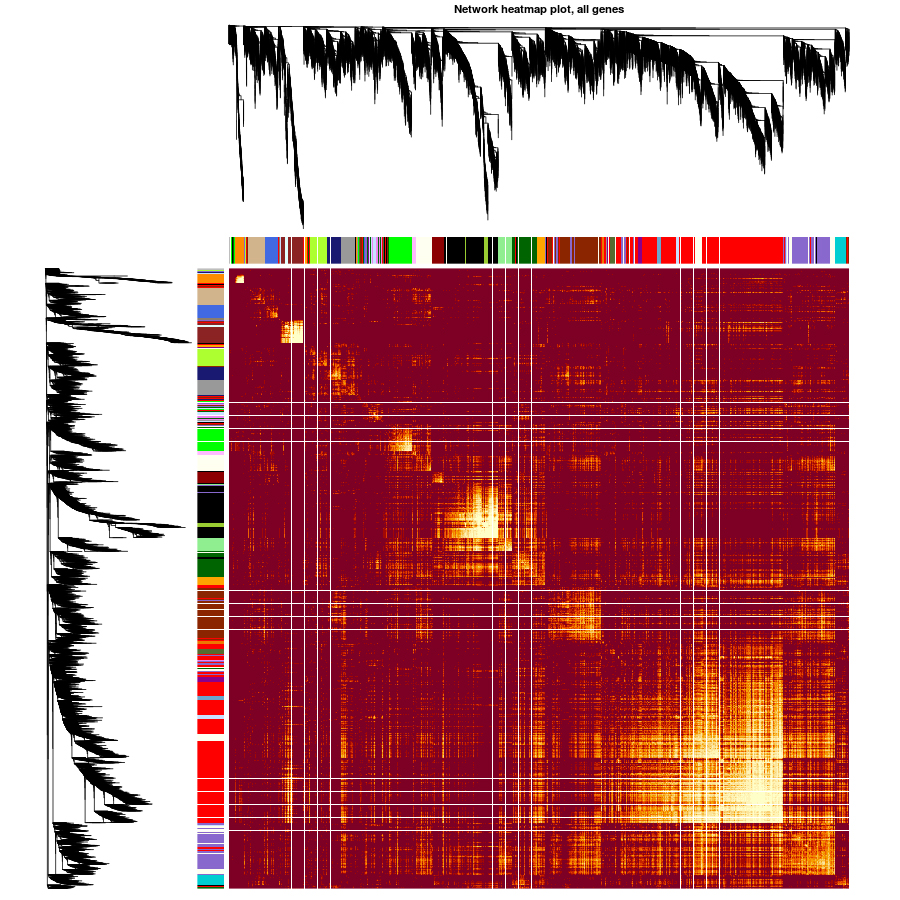

Supplement: Supplementary Figure 1 — The boxplot of Fragments per kilobase of exon per million reads mapped (FPKM) of all samples. [file DataSheet_1.zip › Supplementary Figure S3.jpg]
